# Listerine

**Published:** 1885-01

**Authors:** 


### Listerine.

We have had occasion more than once to call attention to this, to the dentist, highly useful preparation; and it is now again referred to, because there are still many who have not tried it and are ignorant of its value, and there are some who, from sheer neglect, do not use it when and as they ought.

With such an agent as this there is no excuse for offensive odors in or about the office of the dentist. As a deodorant and antiseptic in every place where such agents are required, Listerine stands pre-eminent. While it is equal to any and superior to most of the agents commonly used under such circumstances, it adds an agreeable aroma instead of an offensive odor to the surroundings. It may be freely used in spray or lotion, without stain or irritation, as an agreeable and effectual detergent.

It is also specially commendable, in a week solution, as a mouth wash and gargle for aphthous sores or a fungous condition of the gums and bad breath; and for certain forms of indigestion—those accompanied by disagreeable emetations—a few drops of Listerine in water, swallowed, is a particularly grateful and excellent remedy. Experiment has demonstrated the fact that Listerine compares favorably with the most reliable agents for the rapid destruction of micro-organisms.

This is not a secret preparation, but is made from well-known and pure drugs, the names of which are given upon each and every bottle sent out.

